# The Backfolded Odijk Regime for Wormlike Chains Confined in Rectangular Nanochannels

**DOI:** 10.3390/polym8030079

**Published:** 2016-03-14

**Authors:** Abhiram Muralidhar, Michael J. Quevillon, Kevin D. Dorfman

**Affiliations:** Department of Chemical Engineering and Materials Science, University of Minnesota — Twin Cities, 421 Washington Ave. SE, Minneapolis, MN 55455, USA; mura0122@umn.edu (A.M.); mquevill@nd.edu (M.J.Q.)

**Keywords:** DNA, confinement, genome mapping, nanochannels, Flory theory, backfolding, hairpin, Odijk, rectangular channels, semiflexible polymers

## Abstract

We confirm Odijk’s scaling laws for (i) the average chain extension; (ii) the variance about the average extension; and (iii) the confinement free energy of a wormlike chain confined in a rectangular nanochannel smaller than its chain persistence length through pruned-enriched Rosenbluth method (PERM) simulations of asymptotically long, discrete wormlike chains. In the course of this analysis, we also computed the global persistence length of ideal wormlike chains for the modestly rectangular channels that are used in many experimental systems. The results are relevant to genomic mapping systems that confine DNA in channel sizes around 50 nm, since fabrication constraints generally lead to rectangular cross-sections.

## 1. Introduction

The confinement of a wormlike chain in a channel smaller than its persistence length is commonly referred to as the Odijk regime [1,2]. For short chains in the Odijk regime, the frequency of backfolding is governed by a balance between the enthalpic penalty due to sharply bending the chain and the entropy gain due to the larger configurational space of a folded chain [3]. For long chains, excluded volume interactions between backfolded segments of the chain become important as well [4]. Based on these two ideas, Odijk proposed that long wormlike chains, such as DNA, confined to channels smaller than the persistence length can exist in two different regimes: (i) a “classic” Odijk regime, where the excluded volume effect is strong and the chain is strongly extended; and (ii) a “backfolded” Odijk regime, where the excluded volume is weak and the chain is able to backfold [4]. We have recently provided simulation evidence that strongly supports Odijk’s theory for confinement in square nanochannels [5] and circular tubes [6]. In the present contribution, we provide similar data for rectangular channels. Our work thus completes the validation of the backfolded Odijk regime.

Odijk’s theory [4] for rectangular channels has two important implications for experiments on confined DNA. From a fundamental standpoint, rectangular nanochannels are the key to an efficient experimental strategy, known as confinement spectroscopy, that uses devices with a fixed channel depth and a variable channel width [7,8,9]. Such devices allow one to measure the chain properties over a range of channel sizes on a single device, which is a cost-effective strategy. Perhaps more importantly, these devices allow one to observe the same molecule in many different channel sizes, making them truly single-molecule experiments [9]. From a practical standpoint, rectangular channels often result from limitations in the device fabrication, and the literature is replete with experimental data obtained in rectangular nanochannels [10,11,12,13,14,15,16]. Lithography and etching have a finite precision, and this effect is enhanced as the channel size decreases. In other words, a 200 nm wide by 200 nm deep channel that ends up being 210 nm wide is less of an issue than a 40 nm wide by 40 nm deep channel that ends up being 50 nm wide. Moreover, during device fabrication, it is important that the channels be smooth to avoid DNA adsorption [2,17]. In practice, this often leads to a sacrifice in the exact value of the channel width in exchange for smooth channels.

In the present contribution, we used pruned-enriched Rosenbluth method (PERM) simulations [18,19] of an off-lattice, discrete wormlike chain model [20] to test Odijk’s theory [4] for DNA confined to a rectangular nanochannel. PERM is a biased chain growth method that avoids the attrition problem for self-avoiding random walks by suitably pruning and enriching configurations, thus enabling simulations of extraordinarily long chains, up to 1,000,000 beads for lattice polymers at the θ point [18]. Our implementation of off-lattice PERM, described in detail elsewhere [20,21], allows us to grow chains out to very high molecular weights (circa $10^{4}$ persistence lengths) with high resolution (at least 10 beads per persistence length). Typical simulations here, which sample around $10^{6}$ tours with a maximum molecular weight of approximately $5\times 10^{4}$ beads, require around 13 h on 72 processors for real chains and 1.5 h for ideal chains. Such high molecular weights are key to our analysis. First, they allow us to compute the global persistence length characterizing the bending of ideal chains [5], which is a required input for Odijk’s theory [4]. Second, high molecular weights are required to obtain asymptotically long chains in rectangular channels, since the chain must experience the full effect of confinement from each dimension. We use the simulation data to test Odijk’s predictions [4] for the confinement free energy, average chain extension, and variance about the average extension. At the conclusion of our analysis, we discuss an intriguiging implication of our results for genome mapping technology, as well as the perspectives for using simulations such as ours to test predictions for the blob regimes of confinement in rectangular channels [4,22].

## 2. Odijk Scaling Theory

**Figure 1:**
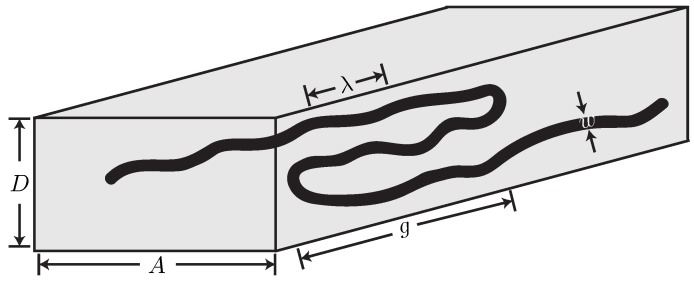
Schematic illustration of a wormlike chain confined in a rectangular nanochannel of depth *D* and width A>D. The global persistence length *g* is the typical distance between hairpin bends, while the deflection segment length *λ* arises from the deflections off the channel walls. The excluded volume is given by a hardcore interaction with width *w* equal to the diameter of the chain. The global persistence length and deflection segment length are not drawn to scale.

The problem at hand, illustrated in Figure 1, consists of a wormlike chain of persistence length $L_{p}$, effective width *w*, and contour length *L* confined in an infinitely long, rectangular channel of height *D* and width *A*. For definiteness, the channel height is the smaller dimension, *i.e.*, D≤A. Odijk [4] describes such channels as nanoslits. This term is used differently in different papers, so it is worthwhile to clarify its definition here. We use the most common nomenclature [23], where a rectangular nanochannel corresponds to values of *A* where the chain feels the effect of confinement of the walls in that direction and a nanoslit corresponds to values of *A* so large that confinement is effectively between parallel plates. In what follows, we only consider rectangular nanochannels and chains with contour length *L* sufficiently long so that the average extension, variance about that extension, and confinement free energy become extensive in contour length. We also only consider channel sizes that correspond to the Odijk regime. Odijk proposed that $D≲\pi L_{p}$ should be the restriction on the channel size [4]; detailed simulations [24] suggest the requirement is closer to $D<4L_{p}$. Here, we use a more conservative condition, $D\leq 2L_{p}$. In the nomenclature of Werner and Mehlig [22], the portions of the phase space we consider correspond to regimes IIIa and IIIb for confinement of a semiflexible polymer in a rectangular channel. Since regime IIIa is the classic Odijk regime, which has been studied extensively in rectangular channels [25,26], our focus will be on the backfolded Odijk regime (regime IIIb).

For channels where $D≲L_{p}$, Odijk’s theory [4] replaces the original wormlike chain with a “chain of deflection segments” [5]. Thus, the chain now consists of $N_{\lambda }=L/\lambda$ segments, where $\lambda ≅D^{2/3}L_{p}^{1/3}$ is the contour length of each deflection segment [1]. Note that the deflection segment length is governed only by the smallest dimension in the channel. The bending of the chain of deflection segments along the axis of the channel is characterized by a global persistence length, *g*, which we will discuss in more detail below. For the moment, we simply point out that *g* is defined in the absence of excluded volume for exactly the same reason that the persistence length $L_{p}$ of a wormlike chain is defined in the absence of excluded volume [3,5].

The free energy of confinement, *F*, of a real chain can be written as

(1) $$F=F^{\circ }+F^{\text{ex}},$$

where $F^{\circ }$ represents the energetic cost to convert a coiled ideal chain in free solution into deflection segments, when the chain is confined between the four walls of the channel [1,5]. The term $F^{\text{ex}}$ accounts for the excess energetic cost involved in confining a real chain. Using a Flory-like approach, the confinement free energy can be written as [4]

(2) $$\frac{F}{k_{B}T}≅\frac{F^{\circ }}{k_{B}T}+\frac{X^{2}}{gL}+\frac{N_{\lambda }^{2}v_{\mathrm{ex}}}{XAD},$$

where $k_{\text{B}}T$ is the thermal energy. The excess free energy, $F^{\text{ex}}$, is split into two parts. The first part, $X^{2}/gL$, represents the entropic cost of stretching an ideal chain of deflection segments to a distance *X* in the nanochannel. The stretching contribution to the free energy is standard Flory theory applied to a chain of deflection segments, which is the reason why the wormlike chain persistence length $L_{p}$ is replaced by the global persistence length *g*. The second part of the excess free energy is the excluded volume arising from $N_{\lambda }$ deflection segments confined inside a volume V=XAD. Odijk computed the excluded volume as [4]

(3) $$v_{\mathrm{ex}}=\lambda^{2}w\langle |\sin\delta |\rangle ,$$

where $\lambda^{2}w$ is the excluded volume between two deflection segments and 〈|sinδ|〉 accounts for the orientation angle *δ* between two deflection segments.

If $D\leq A\leq 2L_{p}$, the leading order contribution to the first term in Equation (2) is given by the free energy of an ideal chain confined in a rectangular channel in the classic Odijk regime [26],

(4) $$\frac{F^{\circ }}{k_{B}T}=\frac{c_{1}L}{L_{p}^{1/3}}\left(\frac{1}{D^{2/3}}+\frac{1}{A^{2/3}}\right),$$

where $c_{1}=1.1036$ is a universal constant [25,26]. In the case of a square channel with size *D*, the right hand side of Equation (4) reduces to $2c_{1}N_{\lambda }$. Note that this part of the free energy was not included in the original Flory theory by Odijk [4], as it has no effect on the average chain extension, but its inclusion here is useful to test the predictions for the excess free energy of confinement. For a rectangular channel with $D\leq A\leq 2L_{p}$, the orientation factor in Equation (3) is approximately [4]

(5) $$\langle |\sin\delta |\rangle \approx {\left(\frac{A}{L_{p}}\right)}^{1/3}.$$

The error in the approximations leading to Equation (5) are provided in supplementary material.

The average extension of the chain is obtained by minimizing Equation (2) with respect to *X*, leading to [4]

(6) $$\langle X\rangle ≃L\xi^{1/3},$$

where

(7) $$\xi \equiv \frac{gv_{\mathrm{ex}}}{\lambda^{2}AD}≅\frac{gw}{A^{2/3}DL_{p}^{1/3}}.$$

Note that there is a typographical error in Equation (19) of Ref. [4] that is corrected here.

The confinement free energy $F_{c}$ corresponds to evaluating Equation (2) at 〈X〉, which produces

(8) $$\frac{F_{c}}{k_{B}T}≃\frac{F^{\circ }}{k_{B}T}+c_{2}\frac{L\xi^{2/3}}{g}.$$

The prefactor $c_{2}$ for the excess free energy is included in Equation (8) to provide correspondence with the ideal chain confinement free energy in Equation (4), where the prefactor $c_{1}$ is known. At the scaling level, the key result is that the excess free energy scaling predicted by Flory theory has the scaling

(9) $$\frac{F^{\text{ex}}}{k_{\text{B}}T}∼\frac{L\xi^{2/3}}{g}.$$

Finally, the effective spring constant is obtained from the second derivative of Equation (2) evaluated at 〈X〉,

(10) $$k_{\mathrm{eff}}={\left(\frac{\partial^{2}F}{\partial X^{2}}\right)}_{X=\langle X\rangle }.$$

From equipartition of energy for a harmonic oscillator, the spring constant is inversely related to the variance of the chain extension about its mean,

(11) $$k_{\mathrm{eff}}=\frac{k_{B}T}{\delta X^{2}}.$$

Combining the latter pair of equations with Equation (2) furnishes the variance in chain extension,

(12) $$\delta X^{2}≅Lg.$$

The scaling laws derived thus far are under the assumption that excluded volume interactions between deflection segments of the chain are weak enough to permit backfolding and hairpin formation. This condition is expressed in scaling form as ${\left(g/\lambda \right)}^{2}v_{\text{ex}}<gAD$, which translates to ξ<1 [4,27]. The key results in Equations (6), (8) and (12) are seemingly identical to those for a square channel in Odijk’s theory [4,5]. The differences are (i) the length scale $D^{-5/3}$ appearing in *ξ* for a square channel of size *D* is partitioned into $A^{-2/3}D^{-1}$ in a rectangular channel and (ii) the global persistence length *g* is different in a square and a rectangular channel. The latter effect is easily seen at a qualitative level. As the aspect ratio increases for a fixed value of *D*, it becomes easier for the chain to bend along the wider direction because the radius of curvature of the hairpin increases. As a result,

(13) $${\left(\frac{\partial g}{\partial A}\right)}_{D}<0.$$

Odijk proposed a mechanical theory for the global persistence length in a rectangular channel, leading to the expression [3,4]

(14) $$g=3.3082\bar{r}\exp\left(\frac{F_{\mathrm{mc}}}{k_{B}T}\right),$$

where

(15) $$\bar{r}=\frac{1.5071L_{p}A}{A+3.0142L_{p}}$$

is the size of the hairpin that minimizes the mechanical energy and

(16) $$\frac{F_{\mathrm{mc}}}{k_{B}T}=\frac{1.5071L_{p}}{\bar{r}}-\ln\left[\left(\frac{A-2\bar{r}}{A}\right)\frac{D}{\pi \bar{r}}\right]+1$$

is the free energy required to bend the hairpin in the mechanical limit [3]. Note that Equations (14)–(16) correspond to those in the more recent paper by Odijk [4].

While our primary objective is to asses Odijk’s scaling theory results in Equations (6), (8) and (12), a necessary first step is to test Odijk’s mechanical theory for the global persistence length in Equation (14). As was the case with our study of Odijk’s scaling theory in square channels [5], the validity (or lack thereof) of Odijk’s theory for the global persistence length is not a prerequisite for assessing the scaling theory. We will compute the values of *g* from simulations of channel-confined ideal chains, which will then be used to analyze the simulations of channel-confined real chains.

## 3. Simulation Method

The simulation method used here is identical to our previous study of the backfolded Odijk regime in square nanochannels [5] with the obvious exception that the channels here are rectangular. In order to verify that our results for rectangular channels reduce to that of squares when the aspect ratio tends to unity, we perform simulations in square channels as well. Briefly, the chain is modeled by N+1 beads connected by rods of bond length *b*, corresponding to a contour length of L=Nb. To incorporate excluded volume effects, we impose a hard-core width of *w* for each bead. For ideal chain simulations, w=0 and for real chain simulations, w=b. We make use of PERM, a chain growth method that natively produces information as a function of contour length. The wormlike nature of the chain is enforced by a bending energy between each trio of contiguous beads, and the beads experience hard-core excluded volume interactions with other beads and the channel walls. Additional technical information about our implementation of PERM [20,21] and its application to confinement in the Odijk regime [5,28] are available elsewhere.

To measure the global persistence length for a particular value of *D* and *A*, we simulate chains with zero excluded volume. We then measure the projection of the mean squared end-to-end distance $R_{x}^{2}$ of these ideal chains along the channel axis as a function of contour length [5]. The global persistence length is extracted by fitting the data with the one-dimensional wormlike chain result [29,30]

(17) $$R_{x}^{2}=\frac{1}{3}\left(1+2m\right)\left[2gL-2g^{2}\left(1-\exp\left(-L/g\right)\right)\right],$$

where

(18) $$m=\frac{1}{2}\left(3\langle \cos^{2}\theta \rangle -1\right)$$

is the orientational order parameter [29,31,32]. In the latter, θ is the angle formed between the tangent to the chain backbone and the channel axis.

In the analysis of real chains, we account for the excluded volume between the chain and the wall by defining an effective channel depth $D_{\mathrm{eff}}=D-w$ and effective channel width $A_{\mathrm{eff}}=A-w$. As a result, the data presented in Section 4.2 correspond to

(19) $$\xi ≅\frac{gw}{A_{\mathrm{eff}}^{2/3}D_{\mathrm{eff}}L_{p}^{1/3}}$$

rather than Equation (7). Likewise, the values of *g* used to characterize the properties of real chains correspond to the effective channel dimensions $g=g(D_{\mathrm{eff}},A_{\mathrm{eff}})$. To test the prediction of Odijk’s theory for the scaling of the average extension and its variance, we use the mean span,

(20) $$X\equiv \langle \underset{i}{\text{max}}\left(x_{i}\right)-\underset{i}{\text{min}}\left(x_{i}\right)\rangle$$

as the metric of extension as this is the quantity that is measured in experimental studies of DNA in nanochannels [2,33]. In the latter equation, $x_{i}$ is the *x* coordinate of the *i*th bead, where the bead index *i* is an integer such that i∈[1,N+1] and the *x*-direction points down the channel axis.

We carried out a large number of simulations for both ideal and real chains for a variety of persistence lengths and channel sizes, *A* and *D*. The details are summarized in Table 1.

## 4. Results

### 4.1. Global Persistence Length

**Figure 2:**
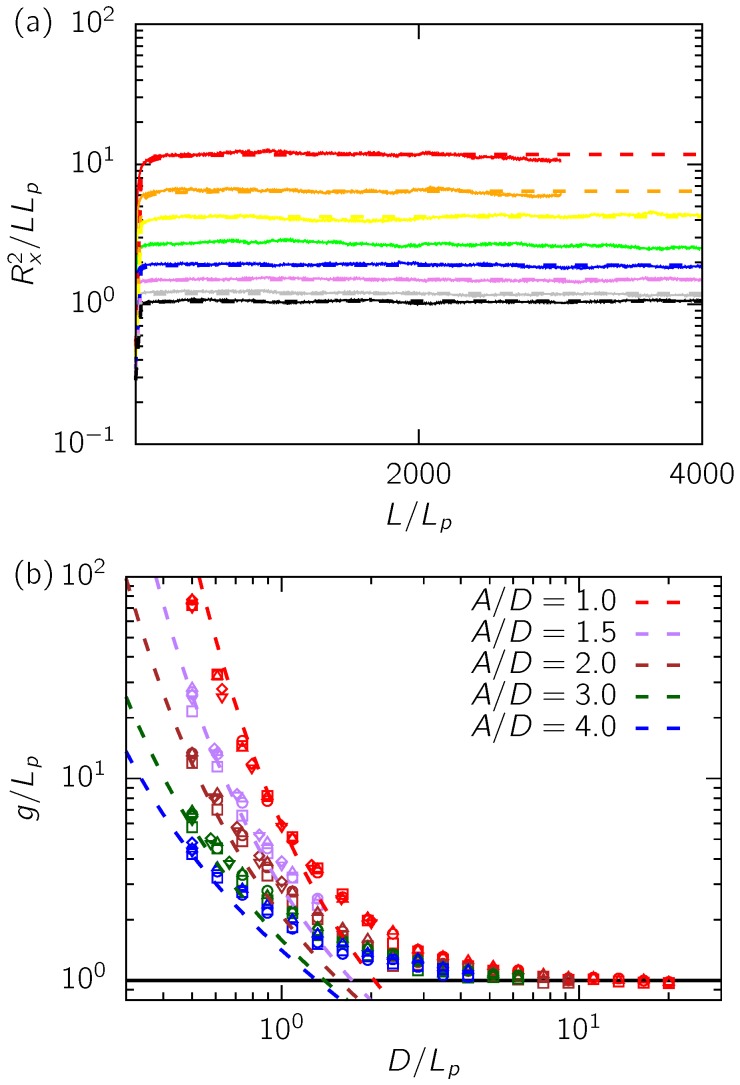
(**a**) Illustration of our method for obtaining the global persistence length. The curves here correspond to a subset of Set 1a (see Table 1) of our data for a fixed aspect ratio of A/D=2 and $L_{p}/b\;=\;10$. The solid curves are our simulation data and the dashed lines are the best fits for Equation (17). The red curve corresponds to the smallest channel size $D/L_{p}=0.5$ and the black curve corresponds to the biggest channel size of $D/L_{p}=1.94$. The channel size increases from top to bottom; (**b**) the global persistence length thus obtained for all our data in Table 1 against dimensionless channel size. The five colors represent the five aspect ratios considered here. The different point types correspond to $L_{p}/b=10$ (□), $L_{p}/b=12.5$ (▽), $L_{p}/b=15$ (◯), $L_{p}/b=17.5$ (⋄) and $L_{p}/b=20$ (△). The dashed lines are from Equation (21). The horizontal black line indicates $g=L_{p}$, which should be the limiting value of *g* for $D\gg L_{p}$.

Figure 2 shows our data for the global persistence length for the entire range of parameters in Set 1 and 2. Figure 2a contains a typical plot of the end-to-end distance along the axis of the channel against the contour length. By using Equation (17), we obtain the global persistence length as a fitting parameter to these curves, as shown in Figure 2a. Figure 2b plots the normalized global persistence length against dimensionless channel size. We observe a collapse of our data for the global persistence length for a fixed aspect ratio of the channel, irrepective of the discretization used in the DWLC models for ideal chains ($L_{p}/b=10,\ldots ,20$). As expected, for a given value of $D/L_{p}$, *g* decreases with increase in *A* in accordance with Equation 13. Furthermore, all the curves in Figure 2b limit to the persistence length ($g\to L_{p}$) for $D\gg L_{p}$, as expected.

To quantitatively assess the dependence of *g* on *A* and *D*, we compare our data to Odijk’s mechanical theory [3]. Odijk derived Equations (14)–(16) in the mechanical limit, neglecting fluctuations of hairpins in the channel. Moreover, the theory makes certain approximations about the shape of the hairpin by considering only the leading order terms to estimate its shape. Cognizant of such approximations, Odijk [3] proposed that there should be corrections to the free energy of hairpins in Equation (16), which can be lumped into an additional term as $F=F_{\mathrm{mc}}+H$. Our comparison with Equations (14)–(16) suggests that the mechanical theory overestimates *g* by about two orders of magnitude (see supplementary material). We find that

(21) $$g=3.3082\bar{r}\exp\left(F_{\mathrm{mc}}/k_{B}T-5.01\right)$$

roughly captures the dependence of *g* on the channel size for all five A/D ratios used in our work. In our previous work on square nanochannels, we found that such a correction leads to good agreement with the simulation data given that $D/L_{p}<1$, which is roughly the range of channel sizes where Odijk’s mechanical theory should be valid. Here, the agreement between this correction for the free energy and its prediction of *g* increasingly deviates from our data as the aspect ratio A/D increases. Note that for a given channel height, $D/L_{p}$, the value of $A/L_{p}$ increases with increasing aspect ratio. Accordingly, the deviation of *g* from the correction to Odijk’s theory for high aspect ratio channels can be attributed to the approximations in Odijk’s mechanical model, which become increasingly worse for larger channels.

### 4.2. Scaling Theory

Although the mechanical theory of Odijk [3] does not quantitatively explain the variation of *g*, this inconsistency does not pose a problem in testing Odijk’s scaling theory [4] as we calculated the value of *g* numerically for a wide range of parameters. Here, we compare our data for real chains to the predictions of the scaling theory by using the numerically obtained values of *g* for equivalent ideal chains, as discussed in Section 4.1. In what follows, we only consider long chains with L≫g, where quantities such as the average extension, its variance and the confinement free energy are extensive in the contour length.

**Figure 3:**
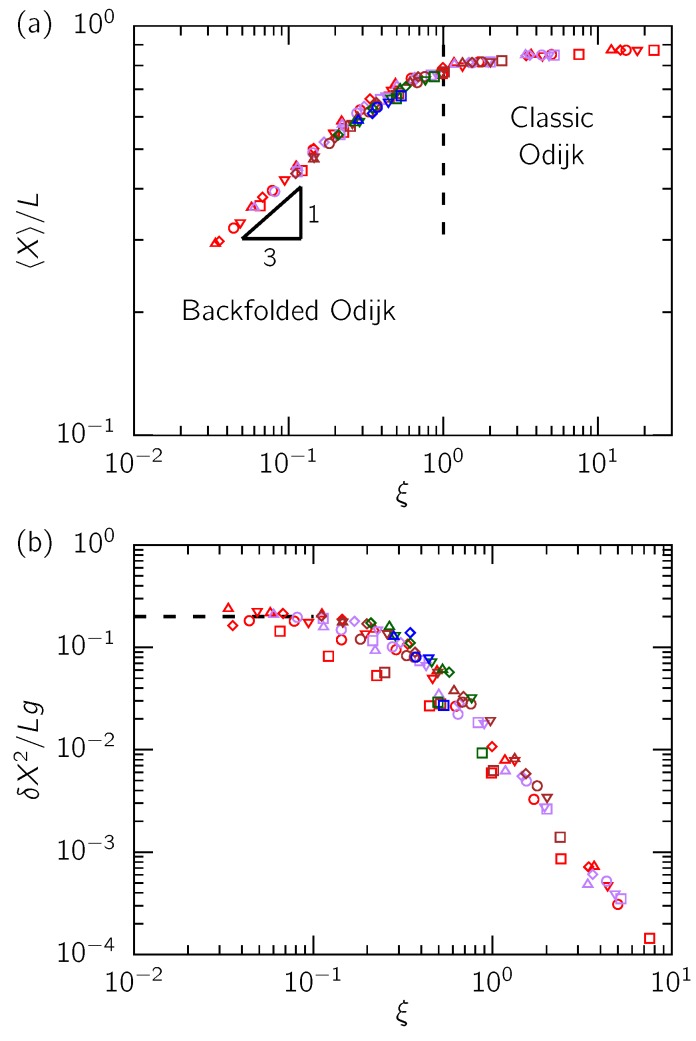
(**a**) Fractional extension against the scaling variable *ξ*. The vertical dashed line shows the boundary between the classic and backfolded Odijk regimes according to the scaling theory, ξ=1. A power law fit to our data for ξ<0.3 yields an exponent of 0.333±0.007; (**b**) normalized variance of extension *versus ξ*. The horizontal dashed line corresponds to $\delta X^{2}/Lg=0.2$. In both the panels, the $L_{p}/w$ values shown are $L_{p}/w=10$ (□), $L_{p}/w=12.5$ (▽), $L_{p}/w=15$ (◯), $L_{p}/w=17.5$ (⋄) and $L_{p}/w=20$ (△). Different colors indicate different $A_{\text{eff}}/D_{\text{eff}}$ ratios: red (1), purple (1.5), brown (2), green (3) and blue (4). All the data points satisfy the condition $D_{\text{eff}}\leq A_{\text{eff}}\leq 2L_{p}$.

We begin our test of the scaling theory by examining the dependence of the average extension of the confined molecule on the scaling variable *ξ* in Figure 3a. This plot was generated by numerically calculating the value of *ξ* from Equation (19) from our ideal chain simulations for equivalent channel sizes $A_{\text{eff}}$ and $D_{\text{eff}}$, similar to our procedure in [5,6]. We observe that our data collapse onto a single curve irrespective of the $L_{p}/w$ and A/D ratios. Indeed, the data exhibit the scaling $\langle X\rangle /L∼\xi^{1/3}$ for ξ≪1, in accordance with Odijk’s scaling theory (Equation (6)). Furthermore, our data transition to fractional extensions close to unity predicted for the classic Odijk regime as *ξ* approaches 1, consistent with our previous simulations in square channels [5].

We consider the variance of extension in Figure 3b. Although the agreement of the variance data with the scaling theory is not as striking as that for the extension, we indeed see that the data collapse onto the scaling prediction, $\delta X^{2}∼Lg$ (Equation (12)). Similar to our findings for square channels [5], this scaling is limited to the parameter range where ξ<0.1. In comparison, the range of data for the mean extension that agree with the scaling theory (Equation (6)) is wider, as shown in Figure 3a. The data for the variance appear to follow $\delta X^{2}\approx 0.20Lg$, a slightly different prefactor from the value 0.25 that we obtained from our simulations for square channels [5]. Considering the error in measuring the variance of extension, and the statistical errors associated with estimating *g*, the agreement observed in Figure 3b is acceptable.

We now turn our attention to the confinement free energy. In order to verify if Flory theory correctly predicts the scaling of the free energy as per Equation (9), we take a two step approach. First, we calculate the confinement free energy $F^{\circ }$ of ideal chains with various $L_{p}/b$ ratios confined in rectangular channels of various sizes and aspect ratios, as summarized in Table 1. In all our calculations, the free energy is computed with a reference state corresponding to an ideal unconfined chain with the same $L_{p}/b$ ratio. Second, we compute the confinement free energy of equivalent real chains, $F_{\text{c}}$, confined in channels with the same values of $A_{\text{eff}}$ and $D_{\text{eff}}$ used for the ideal chain simulations, again using a reference state of an ideal unconfined chain. This allows us to compute the excess free energy as a difference of the two quantities as $F^{\text{ex}}=F_{\text{c}}-F^{\circ }$.

**Figure 4:**
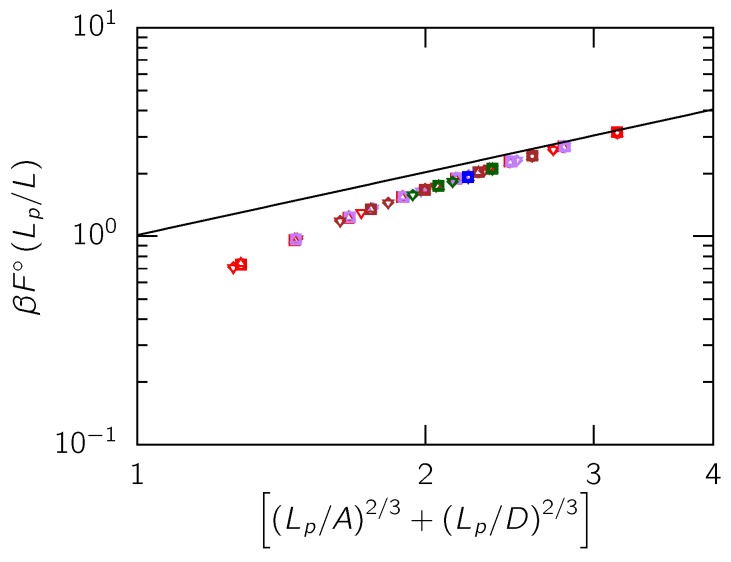
Confinement free energy of ideal chains in various channel sizes. The black solid line is the confinement free energy in the classic Odijk regime valid in the limit $D\ll L_{p}$ and $A\ll L_{p}$ (Equation (4)). The $L_{p}/b$ values for ideal chains shown here are $L_{p}/b=10$ (□), $L_{p}/b=12.5$ (▽), $L_{p}/b=15$ (◯), $L_{p}/b=17.5$ (⋄) and $L_{p}/b=20$ (△). Different colors indicate different A/D ratios: red (1), purple (1.5), brown (2), green (3) and blue (4). All the data points satisfy the condition $D\leq A\leq 2L_{p}$.

Figure 4 shows the confinement free energy of ideal chains confined in rectangular channels, $F^{\circ }$, against a form of the dimensionless channel size suggested by Equation (4). We observe a collapse of our data points irrespective of the aspect ratio of the channel and the channel size indicating that the dimensionless quantity ${\left(L_{p}/A\right)}^{2/3}+{\left(L_{p}/D\right)}^{2/3}\gg 1$ determines the behavior of the free energy in this range of channel sizes, in accordance with Equation (4). Furthermore, in the limit ${\left(L_{p}/A\right)}^{2/3}+{\left(L_{p}/D\right)}^{2/3}\gg 1$ corresponding to the classic Odijk regime, our data collapse onto to the exact solution (Equation (4)) from Burkhardt *et al.* [26], which was obtained by numerically solving a Fokker–Planck type differential equation for the confined chain. As expected, for larger channels where ${\left(L_{p}/A\right)}^{2/3}+{\left(L_{p}/D\right)}^{2/3}\approx 1$, the rescaled free energy increasingly deviates as the sum of the rescaled channel sizes approaches 1.

**Figure 5:**
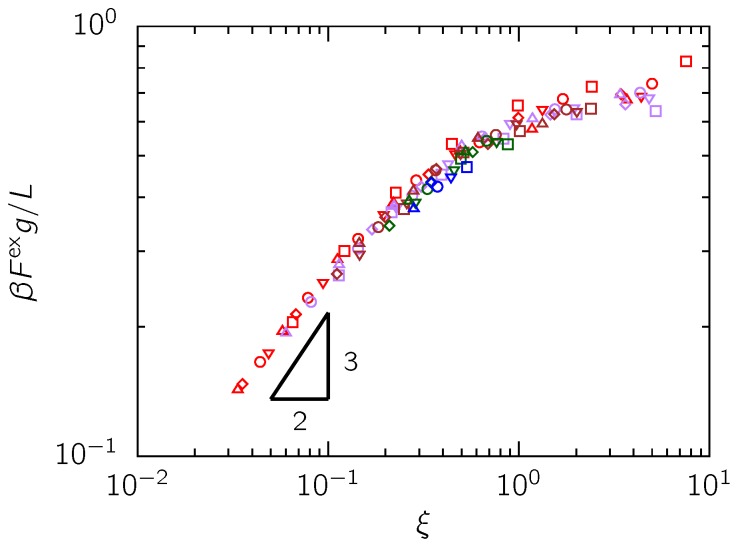
The excess free energy of real chains confined in rectangular channels. The $L_{p}/w$ values shown are $L_{p}/w=10$ (□), $L_{p}/w=12.5$ (▽), $L_{p}/w=15$ (◯), $L_{p}/w=17.5$ (⋄) and $L_{p}/w=20$ (△). Different colors indicate different $A_{\text{eff}}/D_{\text{eff}}$ ratios: red (1), purple (1.5), brown (2), green (3) and blue (4). A power law fit of our data for ξ<0.1 reveals an exponent of 0.559±0.016. All the data points satisfy the condition $D_{\text{eff}}\leq A_{\text{eff}}\leq 2L_{p}$.

To test the scaling prediction of Odijk’s Flory theory in Equation (9), we calculated $F^{\text{ex}}$ by subtracting the ideal-chain contribution from the real chain free energy. Figure 5 shows the rescaled excess free energy plotted against *ξ*. For ξ≪1, we observe a collapse of the data and a scaling of $F^{\text{ex}}∼\xi^{0.56}$ close to $F^{\text{ex}}∼\xi^{2/3}$ predicted by the scaling theory of Odijk [4]. In light of the known drawbacks of the Flory free energy terms for interaction energy and entropy [34,35,36,37], the proximity of the observed scaling to the predicted scaling of the free energy is remarkable. Indeed, Flory theory has been consistently known to incorrectly predict the free energy of real chains in free solution, although the cancelation of errors in the individual terms leads to a good prediction of the scaling of the size of a swollen coil [34,35,36,37]. We suspect that this consistency between Equation (9) and our simulation results is due to the accuracy of the mean field assumptions inherent in Flory theory in the limit ξ≪1, owing to the weakness of excluded volume in the backfolded Odijk regime.

## 5. Discussion

### 5.1. Implication for Genome Mapping

**Figure 6:**
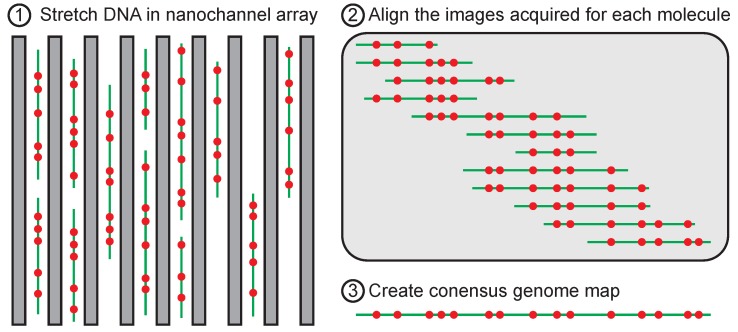
Schematic illustration of genome mapping in nanochannel arrays [38]. The green color corresponds to the fluorescent dye inserted into the DNA backbone, and the red color corresponds to sequence-specific labels inserted into the DNA. The goal of the experiment is to construct a consensus genome map by assembling the measurements of many fragments of the genome that have been stretched in the nanochannel.

In nanochannel-based genome mapping technology, illustrated in Figure 6, DNA are decorated with sequence-specific probes and then injected into a nanochannel. The physical distance between the probes can be converted into a genomic distance provided that (i) the probes are linearly ordered in the channel, so that the order of the probes in the channel is the same as their order along the DNA backbone; and (ii) the stretching of the chain is uniform, so that there is a simple proportionality constant between the physical distance (in nanometers) and the genomic distance (in base pairs). These criteria are met by confining the DNA into a nanochannel that is smaller than the chain’s persistence length, thereby suppressing bending due to thermal fluctuations.

One of the most intriguing aspects of Odijk’s theory is the linear dependence of the variance of the chain extension on the global persistence length in Equation (12). The global persistence length decreases with increasing channel width, but the tight confinement in the channel height maintains the deflection segment behavior. At first glance, it would seem that nanochannel genome mapping could be improved by moving from square nanochannels [38] to rectangular channels of the same depth. For instance, moving from a 50 nm square channel to a 50 nm by 100 nm rectangular channel, the change in extension is only around 30%. This is because the extension would be reduced by ${\left(D/A\right)}^{2/9}{\left(g_{\text{rectangle}}/g_{\text{square}}\right)}^{1/3}$. However, the variance in chain extension is almost halved. Provided that the chain is still sufficiently extended to linearly order the sequence-specific probes on the genome, anisotropic channels may be preferable to their square counterparts by reducing the variance in chain extension.

There is a precedence for using rectangular channels. Indeed, the earliest published data on genome mapping in a nanochannel [39] used 100 nm × 1000 nm slits in a low ionic strength buffer such that the persistence length was similar to the channel depth. The use of wide channels in the latter experiments was motivated primarily by simplifying the fabrication, since such channels are readily fabricated by optical lithography and they can be replicated in poly(dimethyl) siloxane for ease of use in the lab. The introduction of commercially viable devices with 45 nm × 45 nm cross-sections [38] has marginalized the fabrication advantages of the wider slits. Nevertheless, Odijk’s theory suggests rectangular channels may have additional advantages for reducing the error in the genome mapping experiment. This potential advantage needs to be balanced against the reduction in throughput (or, equivalently, the increased device footprint) introduced by wider channels. Moreover, the image analysis may be more complicated in rectangular channels because the DNA can deflect more easily in the wider direction.

### 5.2. Backfolded Odijk Regime for $A>2L_{p}$

In our work, we only considered channels with $D\leq A\leq 2L_{p}$. However, when $D\leq 2L_{p}$ and $A>2L_{p}$, the chain is tightly confined along the side of length *D*, but is free to bend along the other dimension in the channel. Depending on the relative sizes of the sides of the channel, *g* can still be greater than $L_{p}$. One can then derive the scaling of the average extension, the variance of extension and the confinement free energy for such a case using Flory theory along the lines of Equation (2) [4]. The confinement free energy of an ideal chain in this case is dominated by collisions of the chain with the walls along the smallest dimension, *D*, of the channel and can be written as

(22) $$\frac{F^{\circ }}{k_{B}T}=\frac{c_{1}L}{L_{p}^{1/3}}\left(\frac{1}{D^{2/3}}\right).$$

Note that there is a higher order term arising from the collisions of the chain along the larger dimension that scales as $A^{-2}$, analogous to the scaling of the confinement free energy in the blob regimes ($A\gg 2L_{p}$) [20,24,35].

The second term in Equation (2), $X^{2}/gL$, remains unchanged in this case, where the global persistence length, *g*, is again a function of *A* and *D*. However, the weak confinement along the larger dimension causes the deflection segments to be more or less isotropically aligned on an average ($\langle |\sin\delta |\rangle \approx 1$), as in a slit [4,28]. Therefore, $v_{\text{ex}}≃\lambda^{2}w$ (see supplementary material). Minimizing the free energy again with respect to the extension yields the average extension

(23) $$\langle X\rangle ≃L\psi^{1/3},$$

where

(24) $$\psi \equiv \frac{gw}{AD}.$$

The scaling of the variance of extension is unaffected by the change in *A*,

(25) $$\delta X^{2}≃Lg$$

and the excess free energy of confinement scales as

(26) $$\frac{F^{\text{ex}}}{k_{\text{B}}T}≃\frac{L\psi^{2/3}}{g}.$$

The simulation results presented in this paper correspond to relatively modest values of the channel anistropy, ranging from a square channel to a rectangle with A=4D. Unfortunately, testing the scaling laws embodied in Equations (23), (25) and (26) would require simulations in channels with very large aspect ratios (A/D>4) and is computationally challenging. In order to reach the limits where the thermodynamic properties of the confined chain become extensive in contour length, we require chains that are sufficiently long to explore the larger length scale *A*. At the same time, we require that the discretization of the model be sufficiently fine to resolve the chain contour in the smaller length scale *D*. Simultaneously satisfying both constraints necessitates a very large number of beads and thus expensive simulations. While our computational resources would permit somewhat larger values of *A*, it would be very challenging to reach the values A=10D used in the highly anisotropic channels used for some early genome mapping experiments [39] or recent confinement spectroscopy experiments [8]. From a practical perspective, the aspect ratios we studied here are well within expectations for state-of-the-art nanochannel genomic mapping systems [38,40,41] and thus relevant to the design and operation of these devices.

## 6. Conclusions

By performing simulations of asymptotically long chains, we demonstrated that Odijk’s Flory theory correctly captures the mean extension, the variance of extension and free energy for chains confined in rectangular channels for channel sizes where both dimensions are smaller than the persistence length. Our simulations of confined semiflexible polymers in channels with five different aspect ratios show that Odijk’s mechanical theory overestimates the value of the global persistence length by about two orders of magnitude. Nonetheless, the numerical estimation of *g* allowed us to verify the scaling laws over a decade in the scaling variable, *ξ*. The prefactors we obtained for the scaling laws associated with the mean extension, its variance and the confinement free energy can used to predict these quantities for the backfolded Odijk regime in rectangular channels.

The simulation approach we used here could be extended to test the phase diagram proposed by Werner and Mehlig [22] for confinement in rectangular channels in the blob regimes ($D>4L_{p}$). They proposed the existence of seven different regimes of behavior depending on both the smallest length scale *D* and the aspect ratio A/D [22]. While simulating the polymer statistics over this range of channel sizes seems feasible, we again may require very long chains in order to satisfy the strong inequalities that distinguish the different scaling laws. In this case, it may be preferable to switch from our touching-bead model to a bead-rod model, as the latter model has proven effective for simulating wormlike chain confinement in the blob regimes [24]. Our results here complete the verification of the phase diagram for rectangular channels in the backfolded Odijk regime (and others [26] have done equivalent work for the classic Odijk regime), and we are optimistic that the remainder of the phase diagram for confinement in rectangular channels [22] will be verified via simulations in the near future.

## Figures and Tables

**Table 1:** Summary of parameters used in pruned-enriched Rosenbluth method (PERM) simulations. The quantity ${\left[a,b\right]}_{n}$ in the table indicates a sequence of *n* values logarithmically spaced between *a* and *b*, including *a* and *b*. The maximum molecular weight in each simulation was set based on the values of $L_{p}$, *w*, *D* and *A* so as to reach the limit where the theromdynamic quantities are extensive in chain length. Depending on these parameters, the maximum molecular weight in our simulations ranges from 10,000 to 100,000 beads.

		**Confined ideal chains**		
	$L_{p}/b$	$D/L_{p}$	A/D	**# Tours (**$\times 10^{5}$**)**
Set 1a	(10, 15, 20)	${\left[0.5,1.94\right]}_{8}$	(1,2,3,4)	30
Set 1b	(10, 15, 20)	${\left[0.5,1.32\right]}_{6}$	1.5	30
Set 1c	(10, 15, 20)	${\left[2.36,20\right]}_{12}$	2	5
Set 1d	(10, 15, 20)	${\left[2.36,6.23\right]}_{6}$	3	5
Set 1e	(10, 15, 20)	${\left[2.36,4.23\right]}_{4}$	4	5
Set 2a	(12.5, 17.5)	${\left[0.5,2.0\right]}_{7}$	1	60
Set 2b	(12.5, 17.5)	${\left[0.5,1.0\right]}_{5}$	1.5	30
Set 2c	(12.5, 17.5)	${\left[0.5,1.0\right]}_{5}$	2	60
Set 2d	(12.5, 17.5)	${\left[0.5,0.67\right]}_{3}$	3	60
Set 2e	(12.5, 17.5)	0.5	4	60
		**Confined real chains**		
	$L_{p}/w$	$D_{\text{eff}}/L_{p}$	$A_{\text{eff}}/D_{\text{eff}}$	**# Tours (**$\times 10^{5}$**)**
Set 3a	(10, 15, 20)	${\left[0.5,1.94\right]}_{8}$	1	10
Set 3b	(10, 15, 20)	${\left[0.5,1.32\right]}_{6}$	1.5	10
Set 3c	(10, 15, 20)	${\left[0.5,0.90\right]}_{4}$	2	10
Set 3d	(10, 15, 20)	(0.5,0.61)	3	10
Set 3e	(10, 15, 20)	0.5	4	10
Set 4a	(12.5, 17.5)	${\left[0.5,2.0\right]}_{7}$	1	30
Set 4b	(12.5, 17.5)	${\left[0.5,1.0\right]}_{5}$	1.5	10
Set 4c	(12.5, 17.5)	${\left[0.5,1.0\right]}_{5}$	2	30
Set 4d	(12.5, 17.5)	${\left[0.5,0.67\right]}_{3}$	3	30
Set 4e	(12.5, 17.5)	0.5	4	30

## Supplementary Materials

Supplementary materials can be found at www.mdpi.com/2073-4360/8/3/79/s1.

## Abbreviations

The following abbreviations are used in this manuscript:

PERM: Pruned-enriched Rosenbluth method
DNA: Deoxyribonucleic acid
