# Supplementary material for: The Backfolded Odijk Regime for Wormlike Chains Confined in Rectangular Nanochannels
Source: Polymers (Basel). 2016 Mar 14;8(3):79. doi: 10.3390/polym8030079 (PMC6432538; doi:10.3390/polym8030079)
Supplement: Supplementary file 1 [file polymers-08-00079-s001.pdf]

Supplementary Materials: The Backfolded Odijk Regime for Wormlike Chains Confined in Rectangular Nanochannels

Abhiram Muralidhar, Michael J. Quevillon and Kevin D. Dorfman

1. Dependence of the Scaling of Extension on the Aspect Ratio of the Channel

The deflection segment length is governed by the smallest channel dimension D and is given by

$$\lambda \simeq D^{2/3} L_p^{1/3}. \quad \text{(S1)}$$

The excluded volume between deflection segments is

$$v_{\text{ex}} \simeq \lambda^2 w \langle |\sin\delta| \rangle, \quad \text{(S2)}$$

where δ is the angle is between two deflection rodlets. Odijk [4] estimated the scaling of $\langle |\sin\delta| \rangle$ by calculating the average orientation of deflection segments assuming that the confined chain is in a harmonic well with an appropriate potential. The resultant scaling is

$$\langle |\sin\delta| \rangle \simeq \left[\frac{G_D + G_A + 1}{(G_D + 1)(G_A + 1)} \right]^{1/2}. \quad \text{(S3)}$$

If $D \leq A \leq 2L_p$, the two terms in the latter equation are

$$G_D \simeq \left(\frac{L_p}{D} \right)^{2/3} \quad \text{(S4)}$$

and

$$G_A \simeq \left(\frac{L_p}{A} \right)^{2/3}. \quad \text{(S5)}$$

For a square, we have $G_D = G_A$. We further assume that the condition for the Odijk regime leads to $G_D \gg 1$. As a result, the leading-order scaling of $\langle |\sin\delta| \rangle$ for a square channel of size D is given by

$$\langle |\sin\delta| \rangle \cong \left[\frac{2G_D}{G_D^2} \right]^{1/2} \cong \left[\frac{1}{G_D} \right]^{1/2} = \left[\left(\frac{D}{L_p} \right)^{2/3} \right]^{1/2} = \left(\frac{D}{L_p} \right)^{1/3}, \quad \text{(S6)}$$

which is (5) of [4]. For a high aspect ratio channel where $A \gg D$, we have $G_A \ll G_D$. Accordingly, the leading-order behavior is

$$\langle |\sin\delta| \rangle \cong \left[\frac{G_D}{G_D G_A} \right]^{1/2} = G_A^{-1/2} = \left(\frac{A}{L_p} \right)^{1/3}. \quad \text{(S7)}$$

This approximation is used to obtain Equation (5) of the main text and the resultant scaling of extension in Equation (6).

We now compute the errors arising from the approximations in Equation (S7). Assume that $A = nD$. This means that

$$G_D \simeq \left(\frac{nL_p}{A} \right)^{2/3} = n^{2/3} G_A. \quad \text{(S8)}$$

If we continue to consider only the leading-order terms by assuming that the channels are small compared to the persistence length in all cases, then we have

$$\langle|\sin\delta|\rangle \simeq \left[\frac{G_D+G_A}{G_D G_A}\right]^{1/2}. \tag{S9}$$

Substituting for G_D gives

$$\langle|\sin\delta|\rangle \simeq \left[\frac{(n^{2/3}+1)G_A}{n^{2/3}G_A^2}\right]^{1/2}. \tag{S10}$$

We see that $n \gg 1$ is just the large-channel result we derived earlier,

$$\langle|\sin\delta|\rangle \simeq \left[\frac{n^{2/3}G_A}{n^{2/3}G_A^2}\right]^{1/2} = G_A^{-1/2} \simeq \left(\frac{A}{L_p}\right)^{1/3} \tag{S11}$$

If n is not too big, then we have

$$\langle|\sin\delta|\rangle \simeq \left(\sqrt{1+n^{-2/3}}\right) G_A^{-1/2} \tag{S12}$$

This prefactor is not an issue at the scaling level — for the simulations we have the errors in the table below:

$n = A/D$	$\sqrt{1+n^{-2/3}}$
1	1.414
1.5	1.328
2	1.277
3	1.217
4	1.182

Therefore, the approximation

$$\langle|\sin\delta|\rangle \cong \left(\frac{A}{L_p}\right)^{1/3} \tag{S13}$$

has very little effect on the prefactor associated with the scaling of extension in Equation (6) of the main text.

Finally, if $D \leq 2L_p$ and $A > 2L_p$, $G_A \approx 0$. Therefore,

$$\langle|\sin\delta|\rangle \simeq \left[\frac{G_D+1}{G_D+1}\right] \approx 1, \tag{S14}$$

which results in the scaling of extension given in Eq. 23 of the main text.

2. Comparison with Odijk's Theory for Global Persistence Length

Odijk employed a mechanical model to estimate the global persistence length for confinement of ideal wormlike chains in channels with different kinds of cross sections: (i) square (ii) rectangle and (iii) circle [3]. For rectangular channels, the results of Odijk's mechanical model are outlined in Equations (14)–(16) of the main text [3]. In Figure S1, we observe that predictions of the mechanical model (Equations (14)–(16) of main text) overestimates g by about 2 orders of magnitude. This is consistent with what we observed in relation to the mechanical model for confinement in square channels [5] and circular tubes [6].

Odijk derived a similar set of equations that govern the dependence of g on the channel size, D, for confinement in *square* channels as shown below.

$$g = 3.3082\bar{r}\exp\left(F_{\mathrm{mc}}/kT\right), \tag{S15}$$

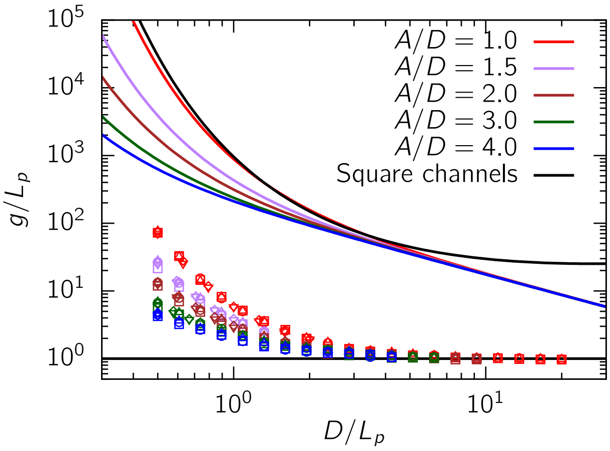

Figure S1. Global persistence length normalized with the native persistence length of the molecule against dimensionless channel size. The five colors (red, purple, brown, green, blue) represent the five aspect ratios considered here. The different point types correspond to $L_p/a = 10$ (□), $L_p/a = 12.5$ (▽), $L_p/a = 15$ (◯), $L_p/a = 17.5$ (⋄) and $L_p/a = 20$ (△). The colored lines are from Equations 14-16 of the main text. The black curve is the prediction for square channels (Equations (S15)-(S17)). The horizontal black line indicates $g = L_p$, which should be the limiting value of g for $D \gg L_p$.

$$\frac{F_{\text{mc}}}{kT} = \frac{1.5071 L_p}{\bar{r}} - 3\ln\left(\frac{D - \bar{r}\sqrt{2}}{D}\right) - \ln\left(\frac{8}{3\pi}\right) \tag{S16}$$

and

$$\bar{r} = \frac{1}{6}\left[\left(2.271 L_p^2 + 9.0426\sqrt{2} L_p D\right)^{1/2} - 1.5071 L_p\right]. \tag{S17}$$

One would expect that the estimate of g in rectangular channels with aspect ratio of unity should reduce to the estimate of g for square channels. To test if this is indeed true, we compare the estimates of g for rectangles with $A/D = 1$ and that for square channels from Equations (S15)-(S17). Although it is not obvious from the equations, we observe that the predictions of the mechanical model for g in square channels and rectangular channels with $A/D = 1$ are almost identical in Figure S1. The two curves for square and rectangle with $A/D = 1$ deviate from each other for $D/L_p > 4$. However, the mechanical model is only valid for $D < L_p$. Therefore, the apparent disagreement between the results for square and rectangular channels for $D > 4L_p$ is unimportant.
